# The effect of sunlight and UV lamp exposure on EPR signals in X-ray irradiated touch screens of mobile phones

**DOI:** 10.1007/s00411-020-00858-7

**Published:** 2020-06-20

**Authors:** Małgorzata Juniewicz, Agnieszka Marciniak, Bartłomiej Ciesielski, Anita Prawdzik-Dampc, Mirosław Sawczak, Piotr Boguś

**Affiliations:** 1 Department of Physics and Biophysics, Medical University of Gdańsk, Dębinki 1, 80-211 Gdańsk, Poland; 2 Department of Oncology and Radiotherapy, Medical University of Gdańsk, Smoluchowskiego 17, 80-214 Gdańsk, Poland; 3 Heat Transfer Department, The Szewalski Institute of Fluid-Flow Machinery Polish Academy of Sciences, Generała Józefa Fiszera 14, 80-231 Gdańsk, Poland

**Keywords:** EPR, ESR, Glass, Mobile phone, Radiation, Retrospective dosimetry

## Abstract

Electron paramagnetic resonance (EPR) signals generated by ionizing radiation in touch-screen glasses have been reported as useful for personal dosimetry in people accidently exposed to ionizing radiation. This article describes the effect of light exposure on EPR spectra of various glasses obtained from mobile phones. This effect can lead to significant inaccuracy of the radiation doses reconstructed by EPR. The EPR signals from samples unexposed and exposed to X-rays and/or to natural and artificial light were numerically separated into three model spectra: those due to background (BG), radiation-induced signal (RIS), and light-induced signal (LIS). Although prolonged exposures of mobile phones to UV light are rather implausible, the article indicates errors underestimating the actual radiation doses in dose reconstruction in glasses exposed to UV light even for low fluences equivalent to several minutes of sunshine, if one neglects the effects of light in applied dosimetric procedures. About 5 min of exposure to sunlight or to light from common UV lamps reduced the intensity of the dosimetric spectral components by 20–60% as compared to non-illuminated samples. This effect strongly limits the achievable accuracy of retrospective dosimetry using EPR in glasses from mobile phones, unless their exposure to light containing a UV component can be excluded or the light-induced reduction in intensity of the RIS can be quantitatively estimated. A method for determination of a correction factor accounting for the perturbing light effects is proposed on basis of the determined relation between the dosimetric signal and intensity of the light-induced signal.

## Introduction

Electron paramagnetic resonance (EPR) signals have been observed in different types of commercial glasses (window glass, windscreen glass, watch glass, glass used for cathode ray tubes, and glass kitchenware) after their exposure to ionising radiation (Engin et al. 2006; Teixeira et al. 2008). The EPR signals generated by doses of a few grays are stable enough to be measured even months after irradiation (Bassinet et al. 2010; Trompier et al. 2011; Juniewicz et al. 2019; Bortolin et al. 2019). Therefore, these materials may be used as potential fortuitous individual dosimeters in radiation accidents. Currently one of the most abundant use of glass worldwide is that for screens of electronic devices, in particular mobile phones. Statistics show that in 2019, the total number of mobile phone users in the world is about 4.7 billion. Screens of mobile phones are especially attractive as radiation detectors not only because of their widespread distribution, but also due to advantages such as chemical inertness, rigidity, insolubility and the fact that a mobile phone is usually kept close to the body, which facilitates estimation of the radiation dose absorbed by its owner. The dosimetric use of screen glasses was already proposed in several reports and articles (e.g., Trompier et al. 2009, Ainsbury et al. 2011).

One of important requirements for reliable retrospective EPR dosimetry is resistance of the dosimeter to other, non-radiation factors, which might affect accurate determination of the intensity of the dosimetric EPR signals, causing errors in dose reconstruction. One of such potential factors is light, both natural and artificial, which is permanently present in human’s environment and has a potential for generation of free radicals. Engin et al. (2006) studied the light sensitivity of window glass samples—unirradiated and irradiated with γ-rays using a 60 W white room fluorescent lamp (up to 8 months) and exposed to daylight (up to 1 year period). No changes in EPR signal were observed in comparison to samples kept in dark for the both types of samples—irradiated and not irradiated. However, in contrast to window glass, the effect of light on EPR spectra was noticed in screen glasses. During an interlaboratory comparison study of retrospective dosimetry using smart phone touch screen glass carried on in 2013, the participants were recommended to expose the irradiated samples to daylight for at least 5 days, to speed up the fading of any unstable EPR signal components (Fattibene et al. 2014). The organizers referred to preliminary findings of the MULTIBIODOSE 2013 report where the light-sensitive component of the EPR spectrum was mentioned. However, the origin of this component and the mechanism and kinetics of its decay after illumination as well as its effect on dosimetry were not described in details.

The results published by McKeever et al. (2019) suggest that the strong background, in both EPR and thermoluminescence (TL) signals observed in some types of mobile phone screens, is caused by their exposure to UV light during production processes. The authors measured a higher intensity of EPR and TL signals along the edge as compared to that in the center of a screen from a Samsung S3 mobile phone, possibly from curing the adhesive between the glass layers by exposure to UV light. Sholom et al. (2019) presented spectra of seven types of paramagnetic centers observed in Gorilla Glass samples—two hole centers (H1 and H2) and five electron centers (E1–E5). Two of them—E2 and E5—were sensitive only to light exposure, while the centers E3 and E4 showed sensitivity to both gamma radiation and light, fading in 6 days after exposure. Sensitivity to light was also proven for other materials used in EPR dosimetry like alanine, where visible light causes fading of radiation-induced radicals, a change in the shape of the spectra, and a decrease in magnitude of the dosimetric signal (Ciesielski et al. 2004, 2008). Also in human nails, generation of a strong EPR signal, similar to the radiation-induced signal, by the UV component of light was recently reported by Marciniak et al. (2019).

In this study, we present effects of exposure of irradiated and un-irradiated mobile phone screen glass to artificial visible light (from fluorescent bulbs), to artificial light including a UV component (from UV lamps used in solaria and cosmetic saloons), and to natural sunlight, on EPR signals of the samples. Consequences of these effects on EPR dosimetry are discussed.

## Materials and methods

### Samples

The samples, each about 90–180 mg in total mass, were obtained from different types of glasses taken from touch screens of mobile phones: Gorilla Glass (marked GG), the type which was also used in the intercomparison study reported by Fattibene et al. 2014; screen glass from iPhone 6S (marked iP_6S); mineral glass from Sony Xperia L, model C2105 (marked MG); and protective screen, a tempered glass (marked TG) used commonly as additional protective cover of the original screen with a thickness of 0.3 mm thickness, and a ninth level of hardness according to the Mohs’ scale. In the periods between the acquisition and crushing of the glass and all subsequent procedures (EPR measurements, irradiations, illuminations), the samples were stored in closed Eppendorf tubes in darkness at room temperature at about 24 °C. After separating the glass screen from LCD layers and after separating the TG plates from the adhesive plastic foil, the samples were washed with ethanol and crushed in a mortar into pieces of approximate shape of elongated triangles or quadrangles with a width of 1–3 mm and a length ranging from 4–22 mm.

### EPR measurements and spectrometer settings

The EPR measurements were performed at room temperature using a Bruker EMX—6/1 spectrometer (Bruker BioSpin) in X-band (9.85 GHz) with a cylindrical cavity of type 4119HS W1/0430. The samples were measured in a quartz EPR tube of 4 mm inner diameter positioned in the central region of the EPR cavity. The cavity was equipped with an internal standard (ER 4119HS-2100 Marker Accessory, Bruker BioSpin GmbH). The EPR acquisition parameters are presented in Table 1. Quantitative analysis of the spectra (alignment and normalization of their amplitudes to the standard’s lines, subtractions of the empty tube spectrum, averaging) was carried out using Microsoft Office Excel 2010.

**Table 1:** Parameters of EPR spectra acquisition

Settings	
Modulation frequency	100 kHz
Modulation amplitude	1.5 G
Microwave power	22.30 mW
Time constant	163.84 ms
Sweep time	83.89 s
Number of scans	10

Numerical fitting/decomposition of the experimental spectra was performed using the Reglinp procedure in Excel with two sets of model spectra: a set denoted as B-R consisting of BG (native background spectrum) and RIS spectra used for all examined types of samples, and a second set consisting of BG, RIS, and LIS (light-induced signal) spectra denoted as B-R-L and used only for the MG and GG glass. The spectra were fitted using g values ranging from *g* = 2.014 to *g* = 1.978 (for TG, MG and iPhone glass) and from *g* = 2.017 to *g* = 1.981 (for the GG samples). The analysis with the B-R set of benchmark spectra is equivalent to ignoring the effects of light, while using the B-R-L set takes into account the light effects in determination of the dosimetric signal. The model BG, RIS, and LIS components were always determined experimentally (separately for each type of the glass samples): the BG was measured in the four types of glass samples neither exposed to ionising radiation nor to light, the model RIS spectra were obtained by subtraction of the spectrum of irradiated (with 10 or 20 Gy), but non-illuminated samples and their BG spectra, while the model LIS spectra were obtained by subtracting spectra of the illuminated, un-irradiated samples and their BG spectra. All those model spectra were determined separately for different types of the glass. The B-R-L decomposition procedure was performed to study the light effects on the BG, RIS, and LIS spectral components in more detail. The results obtained with the B-R procedure, which simulates disregarding in the dosimetric procedures the effects of potential light exposures during normal usage of the phones, allowed to assess how this may affect the numerical values of the reconstructed doses.

To minimize any potential effect of the samples’ anisotropy on the EPR spectra, the measurements were repeated at three orientations of the sample in the cavity, and the resulting three EPR spectra were normalized to the EPR standard’s line, averaged, and normalized to the sample mass. Repeatability of EPR amplitudes at the different orientations of the samples in the cavity was about 7%, uncertainties of the mean amplitudes (standard deviations) were about 4%, uncertainties of the BG, RIS, and LIS spectral components determined by numerical decompositions of the spectra were within 5% of their respective maximum values in each of the figure presented below figures; these uncertainties were not marked in the figures to maintain clarity of the presented data.

### Irradiations

For determination of the dose–response of the EPR signals, the samples were irradiated by 6 MVp photons at the Department of Oncology and Radiotherapy, Medical University of Gdańsk, Poland, using Clinac 600 C/D. The delivered doses were 2 Gy, 4 Gy, 10 Gy and 20 Gy (in terms of absorbed dose to water) involving a dose uncertainty of 2%.

### Illuminations

Three types of light lamps as well as direct sunlight were used for light exposures of the samples. The irradiances of the light sources were measured at the samples positions with an ORION-TH power meter (OPHIR). The artificial light sources were:

1. A lamp made of two parallel CLEO advantage 80 W-R bulbs (Philips) with a power of 80 W each. The total irradiance was 48 W/m^2^.
2. An UV lamp commonly used in cosmetic nail salons for hybrid nail polishing (Ultraviolet Radiant Lamp AP-111, Alle Paznokcie) with four bulbs, 9 W each. The irradiance at the sample position was 164 W/m^2^.
3. A lamp made of six fluorescent bulbs Duluxstar (OSRAM), 24 W each. The irradiance was 110 W/m^2^.

The exposures of the samples to sunlight were done by placing them on a white paper attached to a window sill outside the building at about noon. The irradiance measured during the exposure was about 800 W/m^2^. The total energy exposures (in J/m^2^) were calculated by multiplication of the measured irradiances and the duration of the exposures. The first EPR measurements of the illuminated samples were performed almost immediately (within 0.2 h) after the light exposures and were repeated in the very same samples for several months, as described in the “Results” section.

**Fig. 1:**
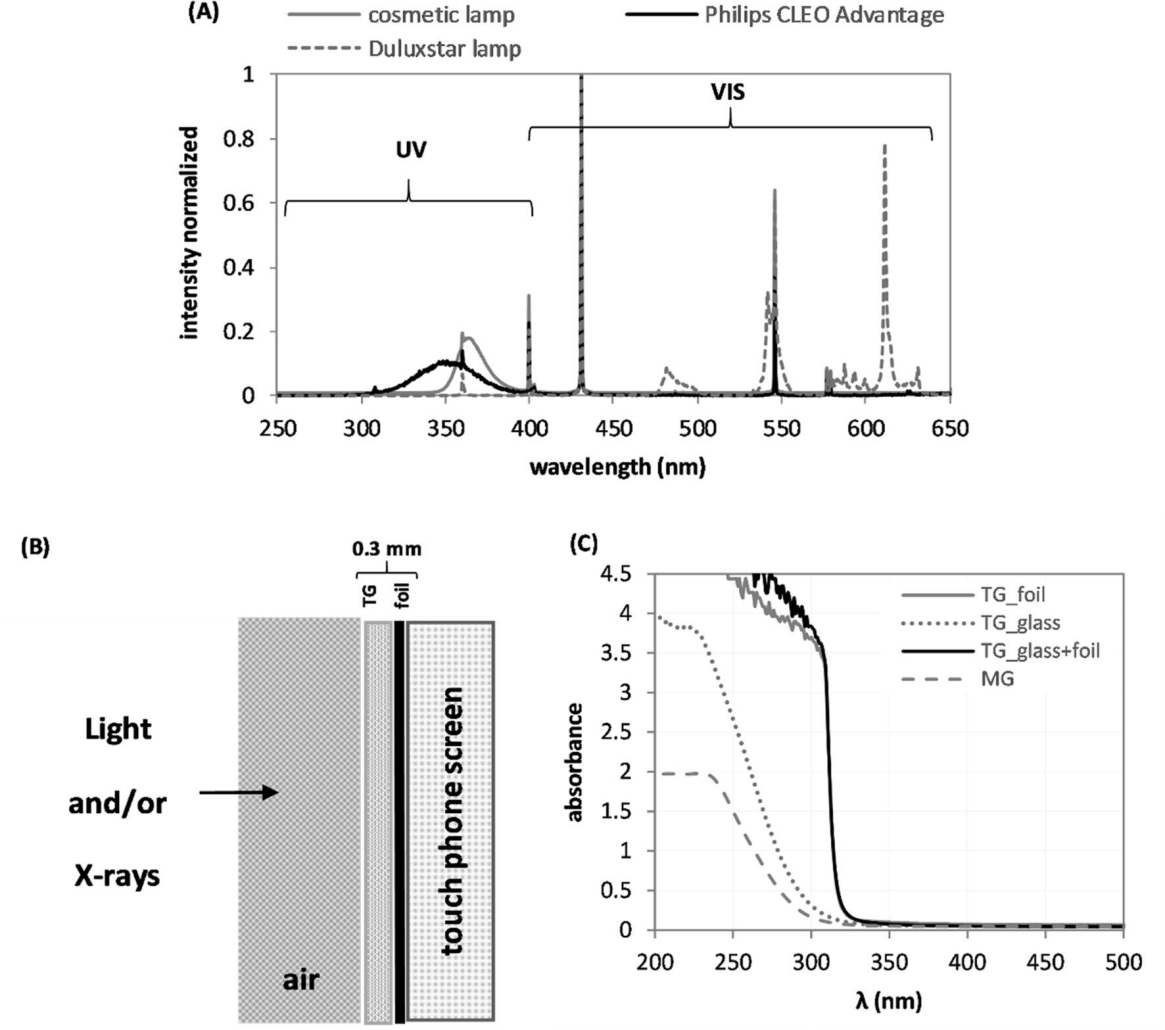
**a** Emission spectra of the Philips CLEO advantage lamp, a cosmetic UV lamp and Duluxstar fluorescent lamp. **b** Schematic representation of the screen layers with the protective layer of tempered glass (TG) and adhesive layer of the plastic foil. **c** The UV–VIS spectra for two types of glass: mineral glass (MG) and TG

Figure 1 shows the light emission spectra for the artificial sources used in this study and UV–VIS spectra for MG and TG. The UV components (below 400 nm) covered about 63% and 72% of the total light intensity, in the cosmetic and Philips CLEO, respectively.

### UV–VIS measurements of glass absorbance

The glass absorbance was measured for layers of TG (Fig. 1b) and for MG with a UV–VIS spectrometer (Lambda 35, Perkin Elmer) in the wavelength range of 200–500 nm. The dependence of the absorbance on the wavelength for the MG and TG is plotted in Fig. 1c.

The data presented in Fig. 1 show that the cosmetic lamp emitted UV light with maximum intensity in the 350–370 nm range, the CLEO lamp in the 350 nm, while the light from the Duluxstar bulbs had only a negligible UV component. The absorbance curves in Fig. 1b demonstrate that neither samples of MG nor TG (with foil) absorb the light emitted by these lamps. Consequently, the protective tempered glass used in this study and/or the adhesive foil do not provide any protection against the radiation-induced effects that might be caused in the screen glass by light with a wavelength of more than 320 nm.

## Results

**Fig. 2:**
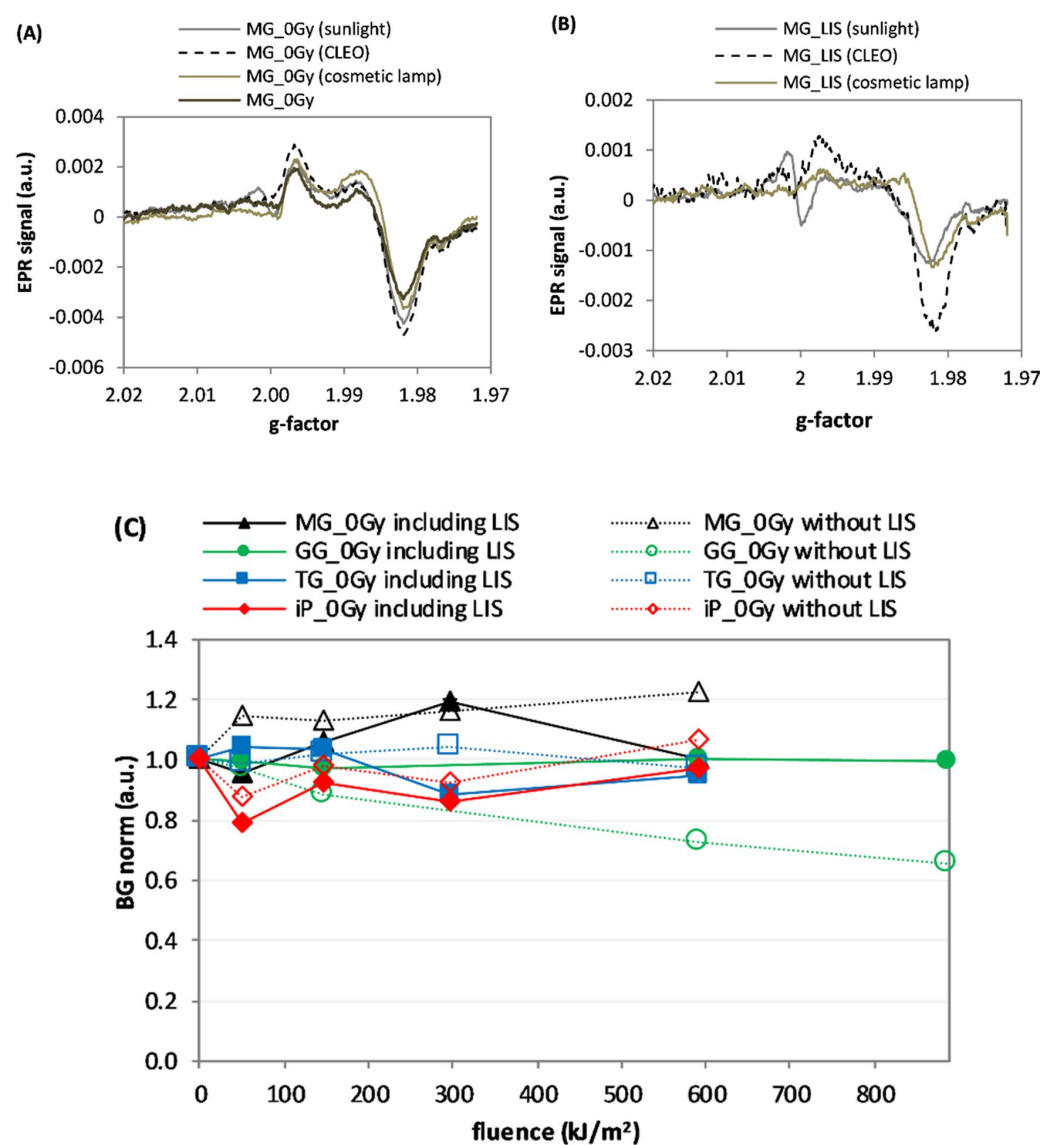
**a** EPR spectra of four mineral glass (MG) samples—one non-illuminated [i.e., the background (BG) signal] and three illuminated with light from three sources including a UV component: direct sunlight, CLEO lamp, and cosmetic lamp with fluences in the range 216–295 kJ/m^2^. **b** light-induced signal (LIS) components in EPR spectra of three un-irradiated MG samples. **c** Comparison of the light effect (for the cosmetic lamp) on the BG components in the samples, determined with the B-R-L and B-R decompositions. Measurement uncertainties are not marked in the figures for the sake of clarity in presentation (for details see text)

The effect of illumination of the un-irradiated MG samples with three types of light including a UV component is presented in Fig. 2. Figure 2a shows changes in the shape of the EPR spectra caused by 5 min of exposure to sunlight, 75 min of exposure to the CLEO lamp, and 30 min of exposure to the cosmetic lamp. The fluences used in these three light exposures were within 216–295 kJ/m^2^. Figure 2b presents the EPR spectra of LIS generated by the three light sources for the MG_0Gy samples. Figure 2c compares the light fluence dependences of the respective BG spectral components in the un-irradiated glasses illuminated with the cosmetic lamp, as determined applying the two decomposition procedures: the B-R-L (i.e., including LIS) and the B-R (without LIS, i.e., ignoring the exposure of the samples to light).

**Fig. 3:**
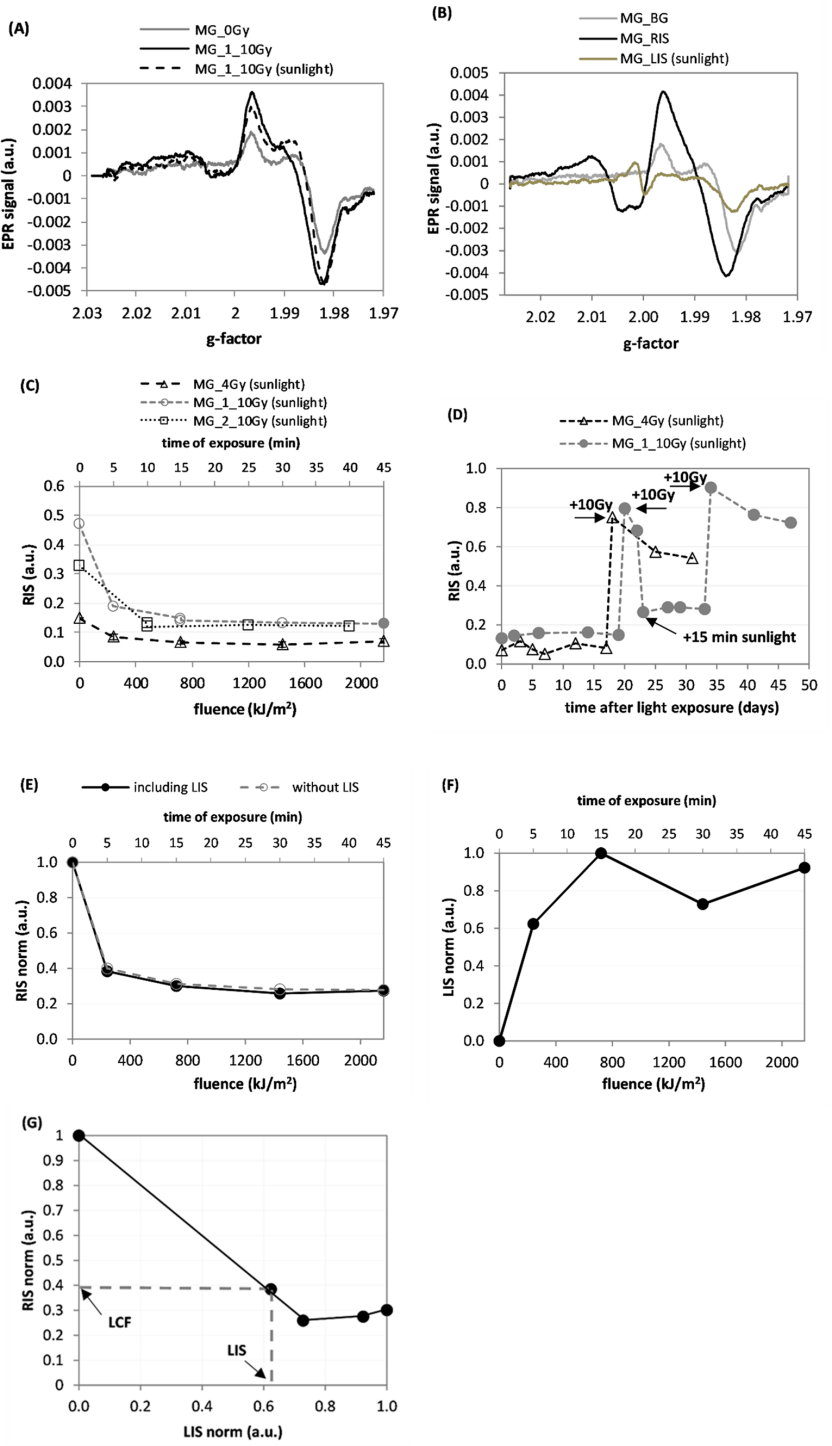
**a** EPR spectra for sample MG_1 after its irradiation to 10 Gy X-rays followed by 45 min exposure to direct sunlight. **b** Three components of EPR spectra for the sample MG_1: background (BG), 20 Gy radiation-induced signal (RIS) and light-induced signal (LIS). **c** Effect of sunlight exposures on magnitude of the RIS component for three MG samples (B-R decomposition). **d** Time evolution of RIS signals for the two samples presented in the (**c**): the MG_4Gy sample was irradiated with an additional dose of 10 Gy on the 17th day after illumination; The sample MG_1 was irradiated with an additional dose of 10 Gy on the 20th day after illumination, and then exposed to 15 min of sunlight on the 22nd day followed by an additional irradiation with a dose of 10 Gy on the 34th day after illumination. These re-irradiations and re-illuminations are marked by arrows. **e** Comparison of RIS vs. light fluence dependences determined by the B-R-L (solid line) or the B-R (disregarding the LIS) decomposition procedure. **f** Effect of sunlight on the LIS component. **g** Dependence of the RIS vs the LIS for sunlight. The dashed lines and the arrows indicate the light correction factor (LCF) determined on basis of the measured LIS component (for details see text). Measurement uncertainties are not marked in the figures for the sake of clarity in presentation (for details see text)

The effects of sunlight on the MG_1 sample irradiated with 10 Gy X-rays are presented in Fig. 3. Figure 3a shows changes in EPR spectra of the MG sample due to its irradiation with 10 Gy and subsequent illumination by sunlight with 216 kJ/m^2^. Figure 3b presents the three spectra contributing to the overall EPR signal: BG, RIS and LIS, measured as described in the “Materials and methods” section. Figure 3c shows the light effects in X-ray-irradiated MG samples, expressed as dependences of their calculated RIS components (using the B-R decomposition) on the sunlight fluence. Before the illuminations, the samples MG_4Gy, MG_1, and MG_2 were irradiated with X-rays to doses of 4 Gy, 10 Gy and 10 Gy, respectively. The scale on the vertical axis shows contributions of the benchmark dosimetric RIS component (obtained from the MG sample irradiated to 20 Gy) to the measured spectra. The evolution of the RIS with time after illumination is shown in Fig. 3d. The arrows marked by ‘ + 10 Gy’ point at rapid increases of the RIS signals in those samples after their additional exposure to 10 Gy of X-rays. The drop in the RIS intensity in the MG_1 sample after an additional illumination by sunlight for 15 min is indicated by the arrow marked by ‘ + 15 min sunlight’. The dependences of the RIS and LIS spectral components on sunlight fluence are presented in panels E and F of Fig. 3, respectively, while Fig. 3g presents the corresponding relation between these two spectral features (RIS vs. LIS).

**Fig. 4:**
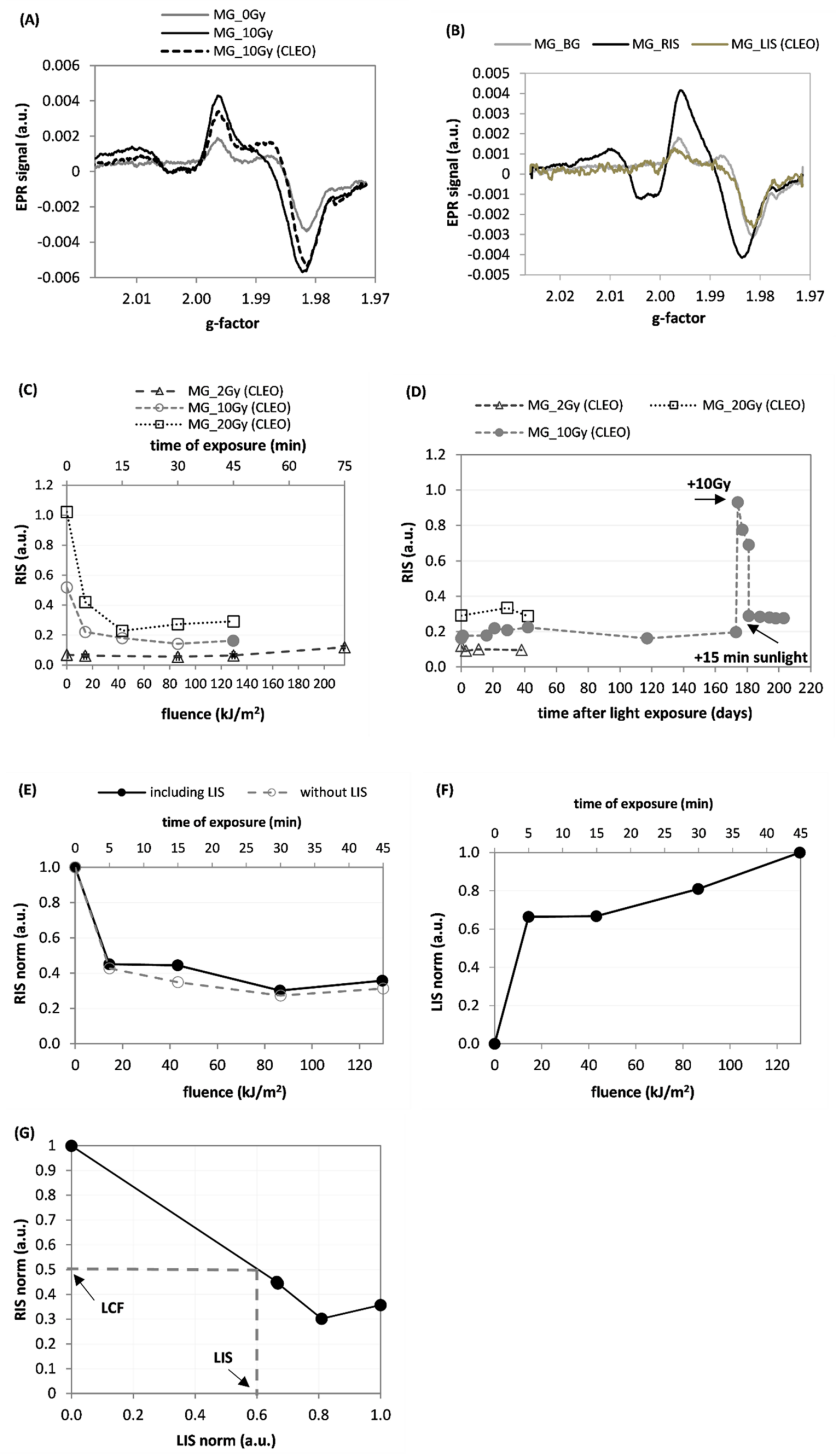
**a** Change of EPR spectra for the MG sample after irradiation to 10 Gy X-rays and then after exposure to the CLEO lamp for 45 min (130 kJ/m^2^). **b** Three components of EPR spectra for the sample MG_10Gy: background signal (BG), 20 Gy radiation-induced signal (RIS) and light-induced signal (LIS). **c** Changes in RIS components (B-R decomposition) after exposure of various doses of X-rays (to 2 Gy, 10 Gy, and 20 Gy) and exposure to light from the CLEO lamp. **d** Time evolution of the RIS for the samples presented in (**c**). The MG_10Gy sample was irradiated at the 173rd day with an additional dose of 10 Gy, and at the 181st day, it was exposed to sunlight for 15 min. **e** Comparison of the radiation-induced (RIS) signal vs. fluence as determined by the B-R-L (solid line) or the B-R (disregarding the LIS) decomposition procedure (dashed line). **f** Dependence of the LIS spectral component in the MG_10Gy sample on fluence of light from the CLEO lamp. **g** Dependence of the RIS on the LIS for the CLEO UV lamp. The dashed lines and the arrows indicate the light correction factor (LCF) determined on the basis of the measured LIS component (for details see text). Measurement uncertainties are not marked in the figures for the sake of clarity in presentation (for details see text)

Figure 4 presents similar data as Fig. 3 but for the MG samples illuminated by the CLEO lamp. Figure 4a shows the spectral changes for the MG sample irradiated with 10 Gy X-rays and then, after 7 days of storage, measured and exposed to the CLEO lamp. Figure 4b shows the three spectra contributing to the overall EPR signal: BG, 20 Gy RIS, and LIS generated by the CLEO lamp. The data presented in Fig. 4c show the light-induced decrease in RIS (calculated using the B-R decomposition) for three MG samples irradiated to 2, 10, and 20 Gy prior to their exposure to the CLEO lamp. Follow-up of the data from Fig. 4c i.e., the time evolution of the RIS in these samples, is presented in Fig. 4d. The sample MG_10Gy was irradiated once more to 10 Gy on the 173rd day after illumination. On the 181th day, this sample was re-illuminated for 15 min with sunlight. Variations of the RIS and LIS vs light fluence are presented in panels (E) and (F) of Fig. 4, respectively, while panel (G) shows the corresponding relation between the RIS and LIS spectral components.

**Fig. 5:**
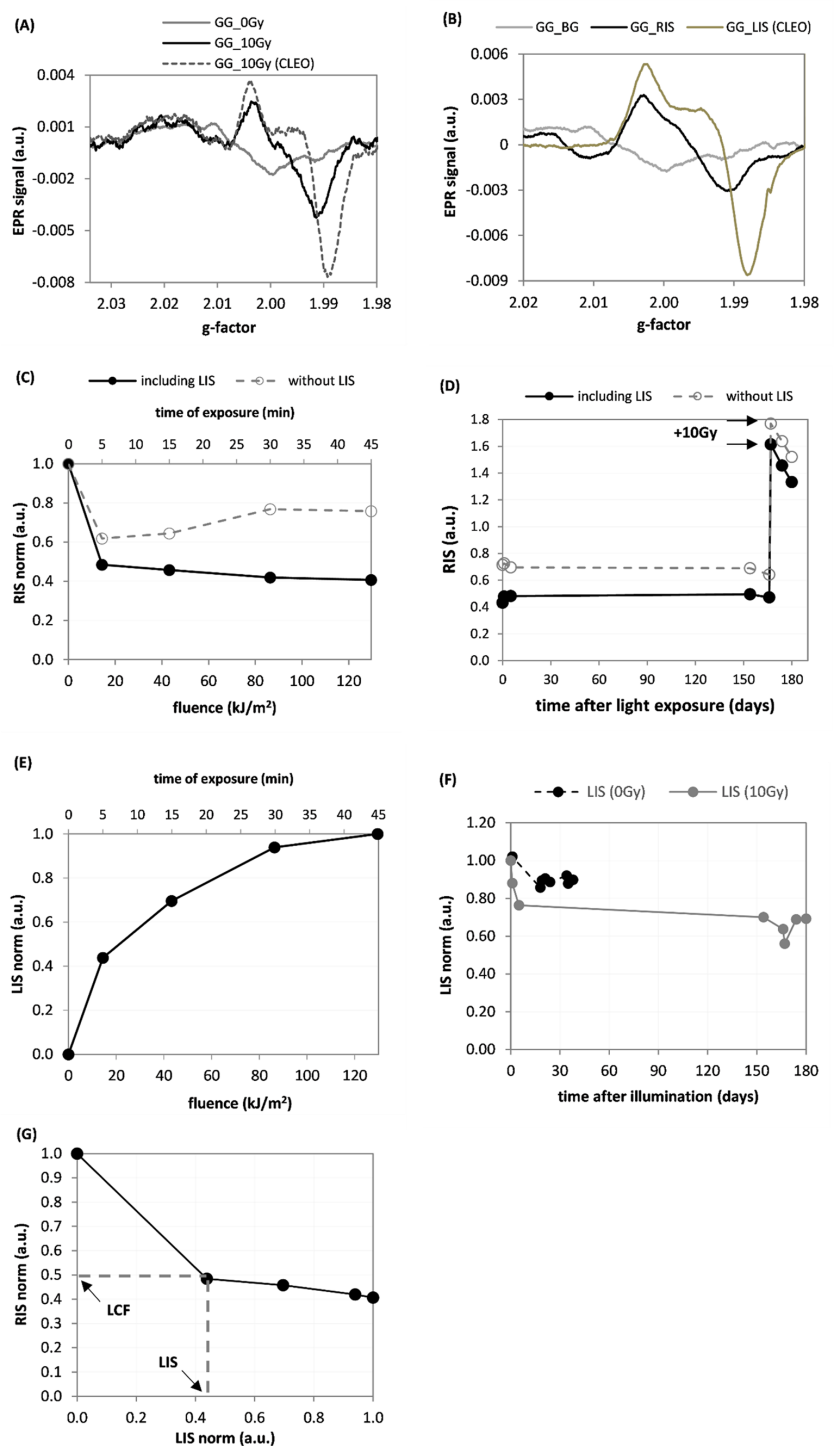
**a** EPR spectra demonstrating the effect of irradiation of the Gorilla Glass (GG) sample with a dose of 10 Gy X-rays, and the effect of a subsequent 45 min illumination with the CLEO UV lamp. **b** Three components of EPR spectra for the GG sample: background signal (BG), radiation-induced signal (RIS), and light-induced signal (LIS). **c** Dependence of the RIS (for the B-R and B-R-L decompositions) on the light fluence from the CLEO UV lamp. **d** Time evolution of the RIS (for the B-R and B-R-L decompositions); on the 166th day, after illumination, this sample was re-irradiated with a dose of 10 Gy. **e** Effect of illumination by the CLEO lamp on the LIS spectral component. **f** Time evolution of the LIS signals in two GG samples: un-irradiated and irradiated with a dose of 10 Gy. **g** Dependence of the RIS on the LIS for the CLEO UV lamp. The dashed lines and the arrows indicate the light correction factor (LCF) determined on the basis of measured LIS component (for details see text). Measurement uncertainties are not marked in the figures for the sake of clarity in presentation (for details see text)

Figure 5a presents changes in the EPR spectra of the GG sample irradiated with 10 Gy X-rays and 23 days later measured, then illuminated with the CLEO UV lamp with a light fluence of 130 kJ/m^2^ and measured again. The light-induced changes in the RIS component determined by both B-R and B-R-L procedures are shown in Fig. 5c. The time evolution of these RIS components is plotted in Fig. 5d; the arrow marked by ‘ + 10 Gy’ shows the increase in magnitude of the RIS components after additional exposure to 10 Gy. Panel (E) illustrates the dependence of the LIS on the light fluence, while panel (F) shows variations of the LIS with time after illumination for the irradiated and un-irradiated GG samples. The decrease in LIS for the 10 Gy sample observed after the 166th day after illumination (Fig. 5f) is probably an artefact resulting from the decomposition procedure: probably a small part of the LIS component was erroneously assigned by the numerical fitting procedure to the spectrally roughly similar RIS component, which strongly increased due to the second irradiation with a dose of 10 Gy on the 166th day.

The light-induced spectral changes in the TG and iP_6S samples did not indicate generation of any specific LIS. In these samples, the light effects were manifested by fading of their RIS components. Therefore, the quantitative analysis for these samples could only be performed using the B-R decomposition procedure.

**Fig. 6:**
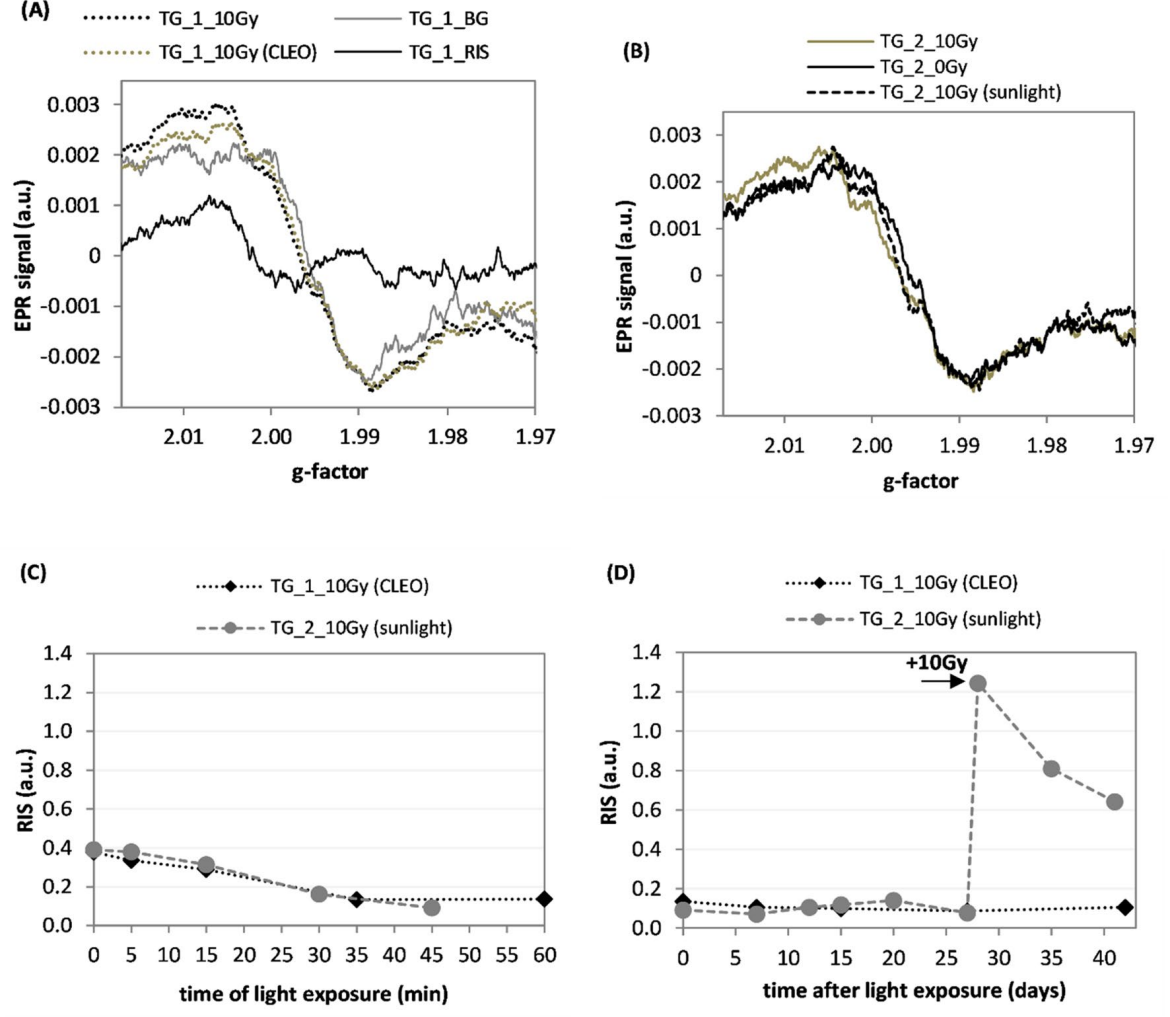
**a** EPR spectra of the TG_1_BG sample—the background signal (0 Gy), 10 Gy radiation-induced signal (TG1_RIS) and the effect of a dose of 10 Gy X-rays and a subsequent 60 min illumination with the CLEO UV lamp [see last data point in p(**c**)]. **b** EPR spectra of the TG_2 sample irradiated with a dose of 10 Gy and then illuminated with direct sunlight. **c** Effect of illumination of the samples TG_1_10Gy and TG_2_10Gy by the CLEO lamp and to sunlight on RIS. **d** Time evolution of the RIS in samples TG_1_10Gy and TG_2_10Gy; the TG_2 sample was re-irradiated with 10 Gy on the 28th day after illumination. Measurement uncertainties are not marked in the figures for the sake of clarity in presentation (for details see text)

Figure 6a and b shows EPR spectra of the TG samples irradiated with a dose of 10 Gy and either illuminated 13 days later by the CLEO UV lamp (light fluence: 173 kJ/m^2^) or 10 days later with direct sunlight (light fluence: 2160 kJ/m^2^). The respective decreases in magnitude of the RIS reconstructed by decomposition of the spectra into their BG and RIS components are presented in Fig. 6c, while the evolution in time of these RIS components is shown in Fig. 6d. The arrow marked by ‘ + 10 Gy’ for the TG_2_10Gy sample indicates generation of the RIS after exposure of this sample to an additional dose of 10 Gy X-rays.

**Fig. 7:**
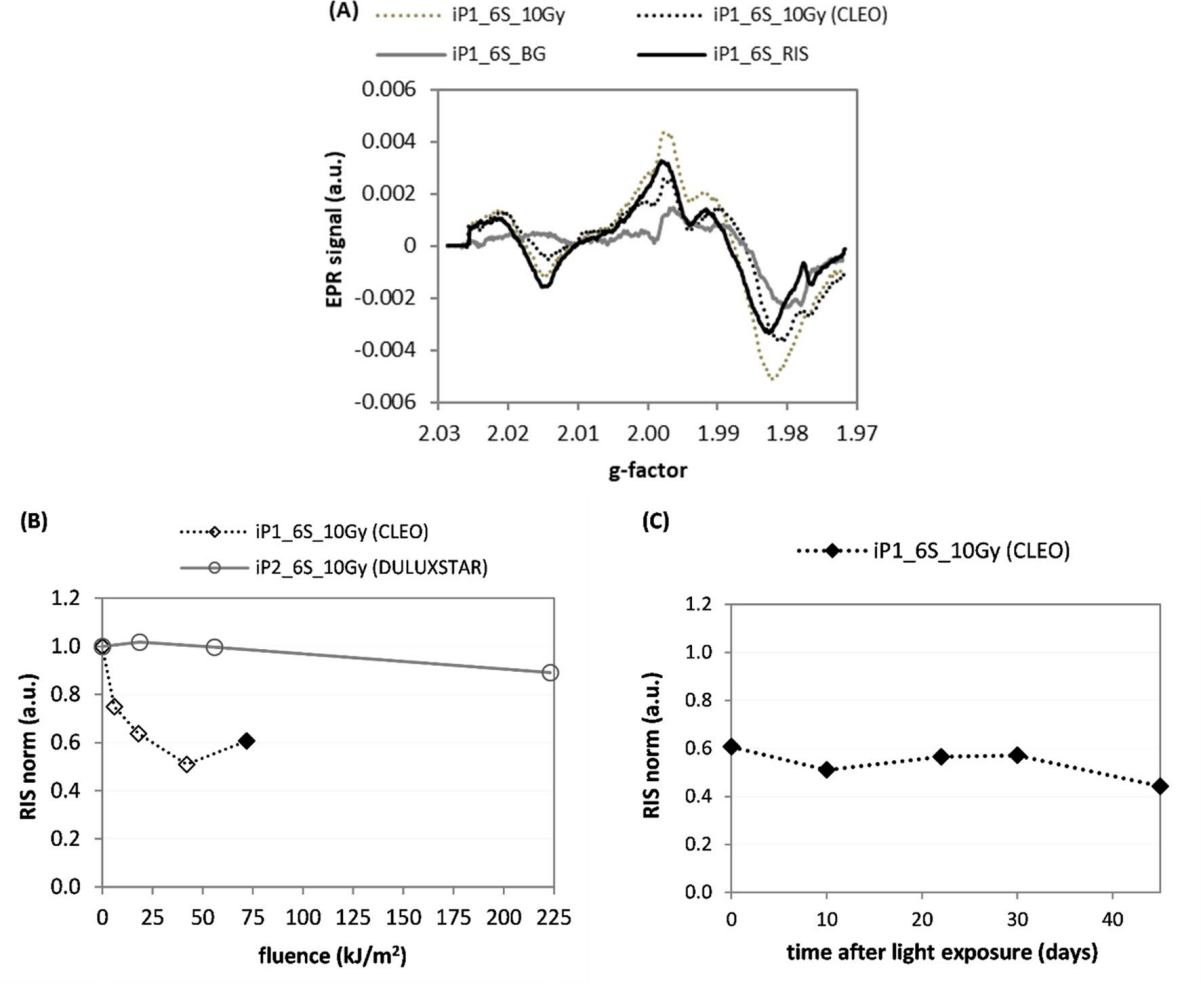
**a** EPR spectra of the iPhone 6S sample—their background signal (BG), 10 Gy RIS and the spectra after exposure to X-rays to a dose of 10 Gy dose, and after illumination by the CLEO UV lamp (for data point marked in **b** by the filled diamond). **b** Effect of light exposure by the CLEO lamp and Duluxstar bulbs on the RIS in irradiated iP_6S. **c** Time evolution of the RIS component in the sample exposed to light from the CLEO lamp (follow-up of the last, black diamond point in (**b**). Measurement uncertainties are not marked in the figures for the sake of clarity in presentation (for details see text)

The effect of irradiation of the iPhone 6S glass samples with a dose of 10 Gy, followed by illumination with the CLEO UV lamp at a light fluence of 173 kJ/m^2^, is presented in Fig. 7a, while the effect of visible light from the Duluxstar bulbs is shown in Fig. 7b. These figure panels compare the effect of exposure to different artificial light sources (Duluxstar bulbs vs CLEO lamp) on RIS (determined by the B-R decomposition), in samples from the same type of mobile phone. In Fig. 7b, the greatest light fluences shown (for the CLEO UV lamp at about 75 kJ/m^2^, for the Duluxstar bulbs at about 225 kJ/m^2^) correspond to illumination times of 60 min. Figure 7c demonstrates the effect of time after the illumination on the RIS component.

## Discussion

Figure 2a demonstrates the changes in the shape of the EPR spectra of un-irradiated mineral glass (MG) caused by exposure to direct sunlight, the CLEO lamp and the cosmetic lamp. The EPR spectra of the illuminated samples, as well the extracted LIS spectra presented in Fig. 2b suggest generation of spectral components by the light for g < 2.00 with a shape similar to that of the native background, and also indicate the presence of an additional paramagnetic center with EPR line at about *g* = 2.00, which was most prominent in the sample exposed to sunlight. Intensities of the BG spectral components in un-irradiated samples did not vary with increase of light fluence from the cosmetic lamp (Fig. 2c), which is particularly evident for the B-R-L decomposition of the spectra.

Analysis of the spectra in Figs. 3a and particularly in 3B shows shapes of the RIS component that are different to those of the BG and LIS components. The initial data points in Fig. 3c (for a light fluence of 0 kJ/m^2^), on the lines showing sunlight-induced decrease in RIS (open squares and circles in Fig. 3c), were measured at different times after X-ray irradiations of those samples. These times were 9 days after irradiation for MG_1 and about 3 months after irradiation for MG_2. The model RIS spectrum used in this analysis was measured in a sample that was exposed to a dose of 20 Gy immediately after irradiation. Different decay of the RIS in these samples explains why the circle and square symbols at 0 kJ/m^2^ light fluence are not at the 0.5 value (which would be expected for samples irradiated with 10 Gy, if no RIS decay occurred). The step-like increases in RIS presented in Figs. 3d and 4d measured after re-irradiation of the samples with an additional dose of 10 Gy, are similar in their magnitudes (about 0.68). This suggests that the radiation sensitivity of these samples (i.e., RIS per unit dose) was not affected by previous light exposures. Also, the decreases in RIS after additional 15 min of exposure to sunlight of the MG_1_10G sample (Fig. 3d) and exposure by the CLEO lamp of the MG_10Gy sample (Fig. 4d) are similar in their magnitudes (about 0.42). This indicates, that the sensitivity of the RIS to light was not affected by previous light exposures of these samples. The light–response curves for RIS and LIS presented in Figs. 3e, f and 4e, f reach a roughly constant minimum value (for RIS) or maximum value (for LIS) after about 5–15 min of light exposure (which corresponded to fluences of about 700 kJ/m^2^ and 20 kJ/m^2^ for sunlight and the CLEO lamp, respectively). The BG and LIS spectra in Figs. 3b and 4b are similar in shape and both are very different from the corresponding RIS (particularly in the spectral region close to *g* = 1.985). This can explain why disregarding the LIS in the numerical decomposition of the EPR spectra of the X-irradiated and then illuminated samples have only minimal influence on the magnitudes of the calculated RIS contributions (Figs. 3e and 4e). Additionally, the contribution from the real LIS in the numerical decomposition of the EPR spectra is accounted for by the BG component, with only a little effect on the reconstructed magnitude of the RIS component.

As can be noticed in Fig. 5a, exposure to X-rays and light causes evident changes in the shape of the EPR lines in the GG samples; the three spectra contributing to the EPR spectrum of the irradiated and illuminated sample, i.e., the BG, RIS, and LIS are very different (Fig. 5b). The light-induced changes in the dosimetric component (RIS) in the GG sample differ significantly when the RIS is determined including or disregarding the presence of LIS in the decomposition procedures (Fig. 5c). This suggests that ignoring the LIS in the decomposition procedures can result in a significant bias on the magnitude of the reconstructed RIS and can cause an overestimation of the actual RIS (i.e., as compared to the situation when the RIS is calculated including the LIS in decomposition procedure) by about 90% for high light fluences. The step-like increase in RIS measured after re-irradiation of the sample with additional 10 Gy (Fig. 5d), is approximately the same as that after the first 10 Gy dose, thus proving, similarly as in the case of the MG and TG samples, that light illuminations prior to X-ray exposure do not affect sensitivity of the GG samples to X-rays. The light–response curve for RIS calculated with the B-R-L decomposition (Fig. 5c) reaches a plateau in the fading after about 5 min of light exposure, whereas the LIS still increases up to about 30–40 min of exposure (Fig. 5e). The magnitude of LIS (Fig. 5f), after a 10–20% drop within the first 1–2 weeks after illumination, was stable over the next 4 weeks (in the un-irradiated sample) and over 6 months (in the sample irradiated with a dose of 10 Gy). The RIS as a function of LIS for the MG and GG samples (Figs. 3g, 4g, and 5g) are of important for practical applications. Namely, they show that the RIS is decreasing with increasing LIS. This relationship may be used to correct the RIS measured in samples exposed to light, thus allowing to minimize the bias in reconstructed radiation doses caused by exposures of the glasses to light. Determination of the LIS component (marked on the abscissa of the RIS vs LIS plots) gives a value for the light correction factor (LCF)—read at the ordinate axis)—as shown in Figs. 3g, 4g, and 5g. The corrected dosimetric signal RIS_cor_ = RIS/LCF can be used for dose reconstruction using an ordinary dose–response curve determined for RIS in a sample not exposed to light. For example, in a MG sample exposed to sunlight with a LIS of 0.62 the LCF is about 0.4 (Fig. 3g), while in a GG sample with a LIS of 0.42 the LCF is about 0.5 (Fig. 5g). Consequently, the corrected magnitudes of the RIS (i.e. which would be measured if the glass was not exposed to light) are 0.62/0.4 = 1.55 and 0.42/0.5 = 0.84, respectively. At lower light fluences resulting in a lower intensity of the LIS spectra, accurate determination of the corresponding LCF requires more data points than measured in the present study, to resolve the trend of the RIS vs. LIS dependence in more detail. The trends used in the present study were obtained only by a rough approximation connecting the two, first left-side points in Figs. 3g, 4g, and 5g, by straight lines. Future studies should also examine how the dependence of RIS on LIS also depends on the time periods between irradiation, illumination, and EPR measurements. This is demonstrated by Fig. 5f showing a decay of the LIS in the first several days after illumination. Further studies are planned for verification and optimization of the proposed correction method and its application in retrospective dosimetry using GG and MG glasses.

Exposures of the X-ray-irradiated TG samples by the CLEO lamp (Fig. 6a) or to sunlight (Fig. 6b) did not induce any significant changes in the shape of their EPR spectra, only a reduction in their intensities. The numerical analysis (i.e., decomposition of the spectra into their corresponding BG and RIS components) proved that illumination of the TG samples caused an about threefold decrease in magnitude of their RIS components (Fig. 6c). The residual RIS in the illuminated TG samples was stable at least for the next four weeks (Fig. 6d). The re-irradiation of the TG_2_10Gy sample with a dose of 10 Gy on the 28th day after illumination caused increase in magnitude of its RIS followed by a decrease of the signal to about 50% in 13 days (Fig. 6d). This result is consistent with those in a previous study (Juniewicz et al. 2019), in which the authors observed a quantitatively similar rate of decay of the RIS components in TG samples. Comparison of the sensitivity to light of the studied samples showed that even a few minutes of exposure of the MG, GG, and iPhone 6S samples to light including a UV component caused a 20–60% decrease of the RIS component (Figs. 3e, 4e, 5c and 7b). It is noted that for the TG glass, this fading was significantly slower than that for the other samples, i.e., an about 50% decay occurred only after 30 min of exposure to light (Fig. 6c).

The RIS signal generated in the iPhone samples was similar to the BG signal, despite the spectral regions at g values of about 1.980–1.985 and 2.02 (Fig. 7a)—these spectral differences apparently were sufficient for the Reglinp procedure to differentiate between the RIS and BG spectral components.

Visible light without a UV component did not cause any evident decrease in the RIS in the illuminated iP1_6S_10Gy sample (Fig. 7b). A lack of any effect of visible light on the EPR signal was also reported by Marciniak et al. (2019) for nails clippings—in their study, the light without any UV component had no effect on the nails’ EPR signal, in contrast to light including a UV component. For the iP1_6S_10Gy sample, the RIS stabilized after the initial light-induced decay and maintained its magnitude about 45 days at about 50% of its value measured shortly after illumination (Fig. 7c).

## Conclusions

The present study showed that in all four types of examined glasses, exposures to light including a UV component (from the CLEO lamp, from a cosmetic lamp, and sunlight) caused significant fading of the dosimetric signal (RIS), which was determined by decomposition of EPR spectra into two separate spectra: background and radiation-induced components. In MG and GG screen glasses, only 5 min of exposure to UV lamps or sunlight was enough to cause a 40–60% reduction of the RIS, while in iP_6S glass this caused an about 20% reduction. The tempered glass (TG) from protective screens was less sensitive to light showing an about 50% reduction in RIS after exposure of about 30 min to the light. Although prolonged exposures of mobile phones to UV are rather implausible, the present results indicated that there is a possibility of underestimating the actual radiation doses in dose reconstruction efforts, in glasses exposed to UV light, if one neglects the discussed effects of light in applied dosimetric procedures. Decomposition procedures performed for the MG and GG samples, taking into account a light-induced reference spectrum (LIS), also showed the light-induced decay of RIS, which in the MG sample was the same for the two procedures. For the GG samples, taking into account the reference LIS spectrum that can be considered as a more appropriate (realistic) and more accurate analytical approach, revealed a much stronger decay in RIS. It is concluded that the light sensitivity of the dosimetric signal can result in a significant bias in retrospectively determined doses. It is emphasized, however, that the present study offers a possibility of quantitative corrections accounting for these effects, based on applying the observed relationship between the LIS and RIS spectral components. This is important, because it has some practical implications in that it improves the accuracy of EPR dosimetry using mobile phone glasses, often being exposed to light in regular everyday use. It is emphasized, however, that this correction can be applied only for glasses, in which light generates LIS that is spectrally different from other EPR signals.
